# Miscellaneous

**Published:** 1874-01

**Authors:** 


### MISCELLANEOUS.

Health at Home; or, Hall's Family Doctor, contains, among a multitude of other practical things, the following articles, adapted to the season and the times:

Frost Bite.—A young lady, after skating some time, complained of one foot being very cold. She was advised by the young gentleman who attended her to put it in warm water as soon as she got home. She did so. The foot inflamed, mortified, and had to be taken off.

It should be remembered that the injury done to a part by burning or freezing is pretty much the same, and is caused by the rapidity of the passage of the particles of caloric, or heat, inwards or outwards. The change should be gradually made; hence, if there is a burn, not deep, it is cured by holding the part near the fire, which is said to draw out the heat. The meaning is, that it is very gradually relieved of the extra heat of the burning. So in frost-bite; go to a room where there is no fire, and rub the frozen parts with snow or cold water, almost ice cold, then rub with flannels, and then with the hands, patiently and rapidly. If a limb is frozen put it in cold water. A frozen part is whitened, hence the cold applications should be made until the color begins to appear. Ice is very brittle, so is anything frozen, the flesh as well, hence a part is easily broken off; therefore handle a frozen part with great delicacy, tenderness and care.

CHILBLAINS are milder forms of freezing, caused by sudden alternations of heat and cold to toes or fingers. There is more itching than pain. Children and old persons—those who are scrofulous or have a feeble circulation—are most subject to them. Bathe the feet in tepid salt water every night. After every washing of the hands wipe them and then rub into them oatmeal or Indian meal so as to effectually dry them. This course of treating the feet and hands tends to keep off chilblains from those who are subject to them.

Wash the blains well three times a day with alcohol or other spirits, or spirits of camphor, rubbing the hands one in another very freely many times a day. A better plan is to take two drachms each of alum and sulphate of zinc to half a pint of water, then add one ounce of spirits of camphor; rub this while warm well into the hands three times a day. If they are broken the ulcers must be treated differently; then use cold water compresses every hour, or every five minutes, until all the inflammation is gone. If there is proud flesh use ointment of red precipitate ointment.

Chilblains are very troublesome, especially in scrofulous and old and feeble persons, caused by the cold inflaming the skin, which assumes a leaden or purple hue. They would never follow cold hands or feet unless they were put in hot water, or held too near the fire. Better to warm them very gradually, by first putting them in cold water, then in water of sixty degrees for five minutes, then rub with the warm hands until they are comfortably warm, using your own hands or those of a more vigorous person.

A Bunion is caused by the pressure or friction of the shoe; the irritation makes the skin swell. Sometimes it is removed in the beginning by keeping a strip of adhesive plaster applied to it as long as there is any discomfort. If inflamed, apply a bread-and-milk poultice, or water compresses, until relieved; or rub into the bunion twice a day, patiently, some ointment made by mixing half an ounce of lard with fifteen grains of iodine, and wear a loose shoe. These bunions generally come on the ball of the great toe, or the inside of the first joint of that toe, or the little toe, or on the instep, caused by too narrow shoes and high heels; hence at once put on a loose slipper without any heels. Cover the bunion with a piece of oiled silk covered with some pain-killer or other ointment. Take a larger piece of buckskin, cut a hole in it large enough to receive the bunion; then another piece of oiled silk over that; in this way the bunion is relieved from the pressure which caused it. Rub the ointment first named on the bunion twice or thrice a day, patiently and well, with the finger.

Sprains in the joints become painful immediately, in consequence of the inflammation. There is one direction applicable to them all: keep down the inflammation by allowing cold water to fall upon it in a constant stream until relief is obtained; as soon as the pain returns repeat the pouring; the colder the water the better; ice water, or salt water which is colder than common water. If it be the ankle, let it be in a horizontal position, so as to favor a return of the blood. If the wrist, keep it elevated a little higher than the elbow, and live on coarse bread and fruits to keep the system cool and open.

Cheap Food.—The following table, taken from Dr. Hall's "Health Tracts" for the people, No. 215, is very suggestive at a time when money is very scarce and provisions are very high.

For those who work, that food is cheapest which, dollar's worth for dollar's worth, affords the most strength, the most power to labor. The investigations and experiments of Baron Liebig and others seem to show that one bushel of oats at sixty-eight cents a bushel, yields five pounds of the muscle, flesh or strength element, costing thirteen cents per pound, while the same amount of "muscle," in the now common acceptation of the term, derived from roast beef, at twenty-five cents per pound at the butcher's stall, would cost two dollars and six cents! The Irish masses do not eat meat once a week, yet they work hard, live healthily, and, when temperate, live long. The Scotch glory in oatmeal, and are a hardy race. One third of the human family live chiefly on rice.

It would be as healthful as economical for the industrious poor of our land to live chiefly on cereals, as wheat, corn, oats, rye and barley, and when they can afford it, have fruits and berries, raw, ripe and perfect in their natural state, as desserts.

| Articles. | Cost. | Muscle element yielded. | Muscle cost per pound. |
|---|---|---|---|
| Oats | $0 68 per bushel, | 5 pounds, | 13 cents. |
| Peas, dried | 2 00 " | 14 " | 14 " |
| Beans, " | 2 50 " | 16 " | 15 " |
| Corn | 1 10 " | 6 " | 17 " |
| Barley | 2 50 " | 8 " | 18 " |
| Turnips | 50 " | 1 " | 41 " |
| Flour, unbolted | 11 00 per barrel, | 25 " | 44 " |
| Flour, fine | 12 00 " | 22 " | 54 " |
| Potatoes | 1 50 per bushel, | 2 " | 94 " |
| Meats, 8½ lbs | 25 per pound, | 1 " | $2 06 " |

Piles Cured.—Unless very aggravated and long neglected, piles may be often cured by avoiding cushioned or other soft and warm seats, and by flapping cold water against the parts until they almost ache with cold, night and morning. This operation contracts the small blood vessels of the parts, which had become relaxed by debility from undue warmth. This contraction drives the accumulated fluids inwards, while the cold bathings give tone, and strength, and vigor to the circulation of the blood all about the adjacent parts.

CORNS.—Soft corns are cured by warm water bathings and buckskin protectors, and no parings are necessary.

Hard corns on the top of the toes, at the joints, can almost always be removed in two or three days by simply soaking the feet in warm water for about twenty minutes, night and morning, rubbing the corn with the end of the finger while under the water. This hastens the softening, and in a day or two the kernel can be picked out with the finger nail. If the corn is shaved off the roots deepen; besides troublesome bleedings sometimes follow, and in several cases have ended fatally. A bit of cotton saturated with oil and bound upon the corn over night, facilitates the softening. The hurting of hard corns before falling weather, is removed by soaking them in warm water.

SAVING FOOD.—About one fifth of the nutritive value of wheat is lost in the bran. This is a clear saving if bread is made out of the whole product of the wheat as it comes from the mill-stones; that is, twenty cents out of every dollar's worth is lost by throwing away the bran. A much larger amount is lost by peeling the common potatoe, as is usually done, for the most nourishing, the part which gives strength to work, is immediately under that thin skin, not thicker than thin paper, and which is peeled off after boiling. The waste in the common paring of potatoes before cooking is a sin and a shame. For delicate stomachs potatoes should be baked, as they are digested in two hours and a half; otherwise, an hour longer; if baked in wood ashes they are sweeter and more healthful.

There is twice as much nutriment in a pound of smoked or dried bacon as in a pound of roast beef.

Roast beef loses fifteen per cent. in roasting; only eleven per cent. if boiled.

A roasted leg of mutton has lost twenty-five per cent.; only ten per cent. if boiled.

The three kinds of food most easily digested are boiled rice, soused pigs' feet and soused tripe, being disposed of in an hour. The following require an hour and a half: Raw eggs, whipped, boiled or fried, salmon or trout, barley soup; sweet apples and venison steak. Roast beef requires three hours and a half, and boiled cabbage and roast pork about five hours.

In any peck of eggs, the difference in weight and nutriment between a dozen of the largest and as many of the smallest is fully one third.

Economy in Fuel.—It is in the matter of fuel that the most cruel frauds are practiced on those who have to purchase in small quantities. In the first place, they have to pay one half more—a dollar and a half for what would cost a dollar by the ton. Every cart load seems black alike, while the amount of actual burning coal in two carts, standing side by side, differs sometimes one half; and it is this inferior coal which is most generally sold by the pail full to the ignorant poor. The flatter the lumps of coal are, the more worthless—being slate, or otherwise "bone," as it is called, because after being burned a while in the grate it turns to a bony whiteness, and comes out as large as when it went in. Good coal leaves nothing in the grate but ashes, and a "scuttle" full, weighing about twenty pounds net, ought not to leave more than two or three pounds of refuse ashes, and all good coal has a "square, shiny feature;" that is, it glistens and breaks in pieces as broad as they are long.

slate and "bone" have a dull look, as if covered with coal dust, and break with difficulty and in thin pieces.

Wood.—There is quite as great a difference in fuel, measure for measure, as in food. One cord of dry hickory will keep up a certain amount of heat for one hundred days; a cord of white oak ninety-one days; while a cord of pitch pine will last only thirty-five days; a ton of coal ninety-one days.

Onions are more economical and more nutritious than is generally supposed. If well dried, four pounds will contain one pound of gluten—an ingredient which gives flour all its value, for without it flour becomes starch, which, as a food, only warms; gives no strength to work.

The Fragments from the tables of New York city would feed abundantly every day a large number of persons. An organization might be formed to district the city, and send a cart to every house in the district once a day to receive the scraps of meat and bread and vegetables which are now thrown into the ash barrel, or unworthily bestowed on persons who are constantly and systematically calling at basement doors as beggars for bread, which is often lavishly used or fed to animals after having been taken home; bread upon which has been plastered in thick layers, butter costing over half a dollar a pound.

Better than Bread!—Some of the organizations in the city prepare delicacies—that is, things which are both digestible and nutritious, for the sick and those who are getting well. But to many who are wearily waiting for health, to prisoners, to the occupants of "Homes," to many who are in lunatic asylums, the reading of a magazine, a newspaper or an illustrated weekly, would many times be better than bread, by diverting the mind and waking up the attention. Pictures will amuse by the hour when the mind is incapable of application, and the heart too much oppressed by sorrow or a hard life. A box of books and old magazines with engravings was sent to a public institution; the Superintendent, in acknowledging the receipt of it, stated that the pictures were sent to the insane department, and that they were looked over with all the greediness and pleasurableness of little children. Could not the "fragment cart" contain a box to receive articles of this kind? There is scarce a family in the city, if arranged with previously, which would not cordially coöperate in these good doings; and many would slip in a dollar or two in money, to supplement a scanty plate of scraps.

Old Clothing.—Many a family has old clothing which it would be glad to get rid of; but when it comes to finding out who it should be sent to, and looking for a messenger or an express wagon to take it, and the half dollar for cartage, it is easy to delay until there be enough to make it more worth while. Meantime weeks and months, or a whole winter, passes by, and what would have literally clothed the naked is devoured by moths. If families could be visited statedly for old clothing, very much good could be done. Many would give cast off garments, or scraps of food, who could not spare money.

Piles, fistulas, fissures, and strictures of various forms, are the results of irregular bodily habits, and many suffer daily tortures for weeks and months without knowing what is the matter with them. William Wood & Co., of Bond street, New York, have published "The Practical Exploration of the Rectum," with an appendix on the "Ligature of Hæmorrhoidal Tumors," by William Boderhamer, A. M., M. D., who, by his ability and skill, has gained the confidence of the entire medical profession. He is the ablest and most successful man living in the treatment of all maladies connected with the pelvic viscera, as will be attested by many of the most eminent men in the United States—Judges, Governors, Congressmen and others.

